# A longitudinal study of risk and protective factors associated with successful transition to secondary school in youth with ADHD: prospective cohort study protocol

**DOI:** 10.1186/s12887-016-0555-4

**Published:** 2016-01-28

**Authors:** Nardia Zendarski, Emma Sciberras, Fiona Mensah, Harriet Hiscock

**Affiliations:** Department of Paediatrics, University of Melbourne, Parkville, 3052 VIC Australia; Community Health Services Research, Murdoch Childrens Research Institute, The Royal Children’s Hospital, Flemington Rd, Parkville, 3052 VIC Australia; Centre for Community Child Health, The Royal Children’s Hospital, 5th floor Flemington Rd, Parkville, 3052 VIC Australia; School of Psychology, Deakin University, Burwood, 3125 VIC Australia

**Keywords:** ADHD, Adolescence, Protocol, Academic achievement, High school, School engagement, Social functioning, Pediatrics

## Abstract

**Background:**

Attention-Deficit/Hyperactivity Disorder (ADHD) has a significant impact on child and adolescent development, especially in relation to school functioning and academic outcomes. Despite the transition to high school being a potentially critical period for children with ADHD, most research in this period has focused on academic outcomes. This study aims to extend previous research by describing academic, school engagement, behaviour and social-emotional outcomes for young people with ADHD in the first and third years of high school and to identify risk and protective factors predictive of differing outcomes across these four domains.

**Methods and design:**

The Moving Up study is a longitudinal, prospective cohort study of children with ADHD as they transition and adjust to high school (age 12–15 years). Data are collected through direct assessment and child, parent and teacher surveys. The primary outcome is academic achievement, obtained by linking to standardised test results. Secondary outcomes include measures of behaviour, ADHD symptoms, school engagement (attitudes and attendance), and social and emotional functioning, including depressive symptoms. The mean performance of the study cohort on each outcome measure will be compared to the population mean for same aged children, using t-tests. Risk and protective factors to be examined using multiple regression include a child, family and school factors know to impact academic and school functioning.

**Discussion:**

The Moving up study is the first Australian study prospectively designed to measure a broad range of student outcomes for children with ADHD during the high school transition period. Examining both current (cross sectional) and earlier childhood (longitudinal) factors gives us the potential to learn more about risk and protective factors associated with school functioning in young people with ADHD. The richness and depth of this information could lead to more targeted and effective interventions that may alter academic and wellbeing trajectories for young people at risk of poor outcomes.

The study is approved by The Royal Children’s Hospital Melbourne Human Research Ethics Committee (33206). Findings will be disseminated through peer-reviewed journals and conference presentations.

## Background

The transition to high school for young people, typically occurring around age 12 to 13 years in Australia, is an important normative life event. Entering high school denotes the end of childhood or the beginning of adolescence. Whilst there is no single definition of the years that constitute the ‘transition’ to high school, it can be conceptualised as the time between the last year of primary schooling and the first 2 to 3 years of senior schooling which, in Australia, lasts six years.

Young people may be apprehensive about moving from the secure and familiar primary (elementary) school environment into an unfamiliar new setting, with the need to establish new relationships with peers and teachers, and meet increased academic demands [1]. It is also a period of rapid physical, emotional and mental changes associated with adolescence and puberty [2]. Despite the challenges it poses, most students transition without too much difficulty, and around 80 % of Australian students go on to complete their final school year [3].

However, the high school transition period does have the potential to alter the education trajectory of individuals and early high school success is important for laying the foundation for future achievement [1]. For a smaller proportion of children, the transition to high school marks a period of declining academic performance, motivation and self-perception [2]. These children are at increased risk of school failure [4] and may begin to disengage from school and ultimately drop-out. Leaving school early has been associated with many adverse consequences including poorer quality of life, lower income, and greater social-emotional problems. Children with neurodevelopmental conditions, such as Attention-Deficit/Hyperactivity Disorder (ADHD), are at increased risk of school failure due to the cognitive, social and behavioural difficulties experienced with the disorder [5, 6].

### ADHD

The Diagnostic and Statistical Manual of Mental Disorders, Fifth Edition, (DSM-5) describes ADHD as a condition affecting children, teens and adults who show persistent and pervasive problems with inattention and or hyperactivity/impulsivity, symptom onset before age 12, and significant impairment in two or more life settings (e.g. school and home). ADHD is estimated to effect 5 % of school aged children and is three times more common in boys than in girls [7]. In about 60–70 % of cases, ADHD symptoms persist beyond childhood to adolescence, however, even when symptoms decline, the impairments associated with ADHD often persist [8]. Young people with ADHD have been shown to have poorer social, cognitive, behavioural and academic functioning in comparison to non-ADHD peers. They remain at significant risk of academic underachievement and poor educational outcomes, and experience lower rates of high school completion, with comparatively fewer completing tertiary education [9].

Young people with ADHD are also at increased risk of experiencing additional mental health and learning disorders. Evidence shows that more than half the children diagnosed with ADHD will experience co-occurring mental health disorders (>60 %) [10–12], the most common of which are internalising (i.e. anxiety and depression) and externalising (i.e. conduct disorders) conditions. Autism Spectrum Disorder (ASD) traits have also been found to be highly prevalent in clinical samples of children with ADHD (30–80 %) [13, 14]. Comorbid learning disorders (math and literacy) (30–70 %) and language and speech problems (12–40 %) are also common [15–17], placing the child at even greater risk of adverse educational outcomes and poorer school functioning [12, 15, 18].

### Transition theory

The critical period when a child enters formal schooling (early years) has been well researched and there is particular focus on ensuring children have the skills and attributes required to start school successfully [19, 20]. Less is known about the transition period from primary to secondary school and a unified theoretical framework has yet to be firmly articulated.

**Fig. 1:**
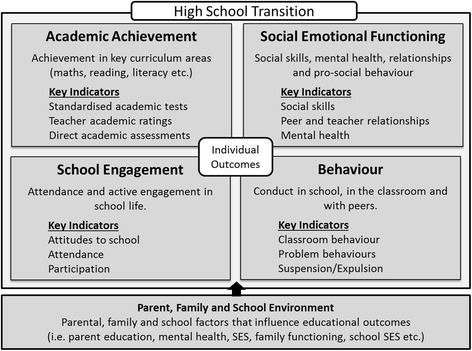
High school transition domains

Exploratory models from studies of middle years education and transitions [21–23] propose models grounded in socio-ecological theory of development [24], to ensure the multiple individual and environmental factors (e.g. parent, family, school factors) are explored. Academic outcomes (school grades, test results) are the most universal measure of school success, however other domains including student wellbeing (social emotional functioning), level of engagement (attendance, attitudes and participation), and behaviour (class conduct and problem behaviours) have been identified as important aspects of school success, and are particularly pertinent transition outcomes [1]. Thus, school transition outcomes should be conceptualised in a number of equally important domains, including academic achievement, social and emotional functioning, school engagement and behaviour [22, 25], as illustrated in Fig. 1.

### The importance of high school transition for children with ADHD

Moving to high school requires young people to quickly adapt to changes in their environment and social settings as they navigate new learning environments, new peer groups, new teachers and different routines. Failure to adapt well is likely to cause increased stress and anxiety, loss of self-esteem and decreased school enjoyment [21]. A negative experience may adversely impact students’ attitudes to school, engagement and academic performance, A successful high school transition experience has been found to protect students, by increasing their connectedness to school and increasing their chance of completing high school [4, 26].

ADHD has a significant impact on child and adolescent development, especially in relation to academic achievement, social skills and school functioning [27, 28]. Studies have shown that even those children that receive medication for ADHD or have received behavioural and educational interventions in childhood, continue to show significant academic and school difficulties in comparison to same age peers without ADHD [29–31]. Adapting to the new school context is likely to be more problematic for children with ADHD. The environmental changes have been associated with a halt in the natural decline of core ADHD symptoms that occurs with age, and thus children with more severe ADHD symptoms prior to the transition are at particular risk of poor transition [32].

Social problems are also more prevalent in children with ADHD [33, 34]. Peer and social problems during the transition period have been linked with poorer school functioning, decreased motivation and increased problem behaviours. On the other hand, feeling connected with peers and engaged in school life has been related to fewer classroom and peer problems, fewer emotional problems and greater pro-social skills [4, 35]. It is easy to see how some children with ADHD may become derailed by their early high school experience, impacting on their academic achievement, behaviour, engagement and well-being and ultimately increasing their risk for low education attainment.

### Outcomes of young people with ADHD during high school transition

There is a large body of research examining academic outcomes for children with ADHD across the lifespan [6, 9, 36]. Multiple studies have shown a significant association between ADHD and academic underachievement [37, 38]. For example, compared to typically developing peers, children and adolescents with ADHD have been consistently found to score lower on academic tests of reading and math and score lower on standard achievement tests [13, 39]. However, most studies investigating academic achievement in this population tend to focus single domains of academic achievement and far fewer studies have examined broader domains including spelling, writing, grammar and punctuation. Furthermore, many studies examine academic achievement in broad age groups (e.g. from 6 to 18) [36], therefore academic outcomes during the crucial high school transition period (i.e. years 6–9) are less clear.

It is also common to measure academic functioning in school settings using a number of other indicators, to assess school-based functioning i.e. attendance, behaviour, grades, grade repetition and early school drop-out. Young people with ADHD have been found to be at increased risk of poorer school functioning across all such measures [9]. A recent study of adolescent males in years 9 to 12 (n = 326), found that in addition to poor academic achievement, students were eight times more likely to drop out of school altogether than peers [40]. Relatively few studies however, have investigated predictors of academic achievement and school functioning beyond ADHD symptoms, and more importantly few studies highlight factors associated with academic success. The Pittsburgh ADHD Longitudinal Study (PALS), found that while on average the ADHD group achieved lower academic results and had more academic problems, 30 % of the group went on to enrol in a 4 year tertiary degree. How this group differed from ADHD peers who did not go on to attend university has not been explored.

### Predictors of good high school transition

The aetiology of school functioning problems in children and adolescents with ADHD is likely to be multifactorial including child, parent/family and school factors [6, 10, 41]. Poorer academic performance in young children has been associated with more severe inattention and hyperactivity-impulsivity symptoms, as rated by teachers or parents [6, 40], decreased student motivation, and poorer cognitive abilities, including lower intelligence levels (IQ) and poorer executive functioning and working memory [6, 42]. Furthermore, studies have shown that early externalising symptoms (e.g. aggressive behaviour) and other comorbid mental health conditions are associated with poorer academic functioning in primary school children with ADHD [43, 44].

There are also a number of individual factors that have been found to be more prevalent in children with ADHD and associated with poorer academic and educational outcomes. These include: problems with peer relations including peer victimisation [45], sleep problems [46], irritability [47], cognitive problems [41, 48, 49], working memory issues [49, 50], substance use [51] and delinquency [52], which are all likely to be risk factors for an unsuccessful high school transition. Those factors that are modifiable merit particular focus [53] as earlier and more effective interventions that aim to decrease these factors may mediate the impact of ADHD on high school outcomes.

Student education outcomes in the general population can be influenced by a range of socio-demographic and environmental factors. For example social disadvantage and poverty has been found to adversely affect student achievement and students with parents who have mental health problems are more likely to have worse educational outcomes compared to same aged peers [54, 55]. These factors may also influence school transition outcomes for children with ADHD. There is some evidence, although inconclusive, that secondary school characteristics, such as school sector, location, size and school socio-economic rating may play a role in education attainment [56], although these factors remain unexplored as risk or protective factors one early high school success.

The transition to high school is a critical period and has the potential to alter future academic, educational and consequently, life outcomes. Young people with ADHD are likely to experience a poorer transition, however, few studies have investigated the academic outcomes during this time period, and the predictors of academic achievement remain unclear. Furthermore, even less research has examined how children with ADHD are faring in relation to other important transition domains (school engagement, social and emotional well-being and behaviour) during this time period and the factors that influence better or worse transition outcomes.

### Study aims

This study aims to describe the secondary school transition and early high school adjustment in an established cohort of children with paediatrician-diagnosed ADHD. We will examine how these children are faring across the educational domains of academic achievement, social, behavioural and school engagement in years 7 (first year of high school) and 9 (third year of high school), as compared to the published national student average. Secondly we aim to examine the risk and protective factors that may be predictive of individual transition outcomes.

We hypothesise that young people in years 7 and 9 with ADHD will have poorer outcomes across all transition domains when compared to peers. We anticipate that outcomes (depending on the domain being examined) will be predicted by a range of child factors including ADHD symptoms, comorbid conditions, cognitive ability as well as other child, family (e.g. parent education and mental health), school (e.g. school type and size) and socio-demographic (e.g. age, gender, family income) factors.

## Methods and design

The Moving Up study is a longitudinal, prospective cohort study of children diagnosed with ADHD and recruited in 2014/15. This study is being undertaken by the Murdoch Childrens Research Institute (MCRI).

Participants will be drawn from two existing ADHD cohort studies, namely the Sleeping Sound with ADHD Randomised Controlled Trial (SS RCT, HREC #30033) and the Attention to Sleep (ATS) cohort (HREC # 31193A). The study protocols have been harmonised to ensure consistency in data collection methods and study measures and the methods for each study have been published elsewhere [46, 57].

The children, aged 5–13 years at baseline, were recruited from public and private paediatric clinics (*N* = 21) across the state of Victoria, Australia and met the full DSM-IV criteria for ADHD at the time of recruitment. Diagnosis was confirmed by independent researchers using the ADHD Rating Scale IV and study designed questions to ensure symptoms were present for at least 6 months, with impairment in two or more settings and onset before the age of 7 [58].

Participant families from the original ADHD study cohorts (SS RCT and ATS) have been contacted to confirm eligibility (school year), update contact details and to assess interest prior to recruitment. Children who are in years 7 and 9 (11–15 years) are eligible to participate in the Moving Up study (n = 238), and they are being recruited in two waves (Wave 1: 2014; and Wave 2: 2015).

### Recruitment and consent

An invitation letter has been sent to eligible families describing the study and participant requirements. This letter contains an opt-out slip and instructions on how families can elect to opt-out of the study. After ten days, families who have not opted out of the study are sent an information statement and consent form, which outlines in detail what participating in the study will involve. There are separate forms for the parent/guardian and child. Informed consent is obtained in writing from the parent or guardian and from the participating child (subject to level of maturity, as determined by the researcher), prior to the commencement of the home visit.

A week after sending the information and consent materials to families, the parent/guardian is called to discuss the study information and to invite interested families to enrol in the study. A home visit is scheduled with families that wish to proceed and parents are asked to rate their child’s current ADHD symptoms (off medication) using the established baseline procedure described above, to obtain current ADHD status on entry to Moving Up.

#### Inclusion criteria

Families from the previous ADHD cohort studies, described above, were invited to participate (n = 202) in the follow up if the study child (aged 12–15) was commencing year 7 or year 9 in 2014 or 2015. Children in alternate education settings (i.e. children being home schooled or in special education settings) or children who refuse to attend school (who would otherwise be in year 7 or 9) are included. Participant recruitment is aligned with the National Assessment Program – Literacy and Numeracy (NAPLAN), which is conducted annually in high school setting for years 7 and 9 only.

#### Exclusion criteria

At baseline participants were excluded if the child had a major illness (e.g., severe cerebral palsy) or an intellectual disability (i.e., IQ < 70). Families were also excluded if the primary caregiver did not have sufficient English to complete the surveys. Given the initial focus on child sleep in both studies, children (n = 25) were excluded if they screened positive for obstructive sleep apnoea, assessed using the obstructive sleep apnoea scale from the Children’s Sleep Habits Questionnaire (CSHQ) and telephone consultation with a general paediatrician (HH) [59].

Families that have withdrawn from the original cohort study or who have subsequently indicated they do not wish to take part in future research were not contacted about this study.

### Data collection

**Fig. 2:**
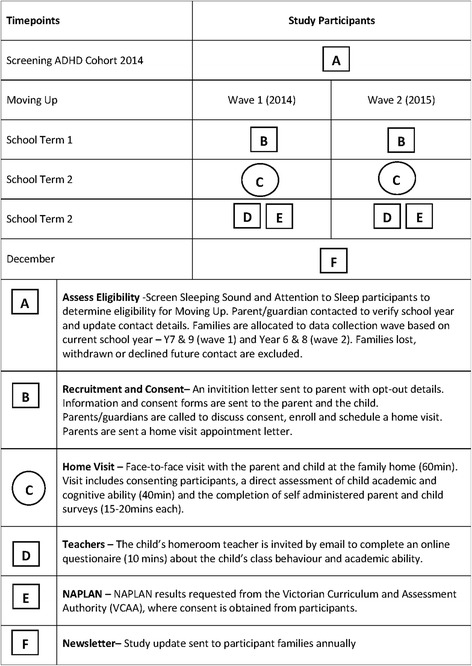
Graphical summary of study design

Data are collected through direct assessments and child, parent and teacher surveys completed using a tablet device (parent and child) or by secure web link (teachers) and through data linkage to standardised academic assessments. A graphical summary of the study design is shown in Fig. 2.

Home visits are scheduled with participating children and their parent/guardian during the second school term, to allow transient issues related to starting a new school to settle. Teachers will be invited to complete teacher surveys in term 3, to ensure all teachers are reporting in the same period and that they have access to midyear reports. Standardised assessments (NAPLAN) are conducted annually at the end of May (term 2) and results are available in October of the test year (term 4).

### Measures

#### Primary outcome

The primary outcome is academic achievement, as measured using standardised achievement tests. Standardised academic testing in Australia (NAPLAN) is conducted annually for students in years 3, 5, 7 and 9. Tests are conducted across five key learning domains: reading, writing, language conventions (spelling, grammar and punctuation) and numeracy. NAPLAN results provide a measure of the students’ academic performance at a point in time, as compared to other students in the state in the same year. A scaled score and a band level are provided for each domain completed by each child. There are 10 band levels, covering the breadth of student achievement. Six of the bands are used for reporting student performance at each year level. For example, the year 7 results are reported across band levels 4 to 9 and year 9 are 5 to 10. The bottom band (i.e. 4 in year 7) denotes children with a score on the learning domain which places them below the national minimum standard (the minimum skill level required for that year) and are at increased risk of academic failure [60]. NAPLAN results, with parent consent, will be sourced from the Victorian Curriculum and Assessment Authority (VCAA).

#### Secondary outcomes

Secondary measures, as listed in Table 1, include a broad range of measures including other measures of child academic achievement, behaviour, social and emotional functioning and student engagement. All measures are well validated for use with children and adolescents and have reliable normative or population data available for comparison to children in the study cohort.

**Table 1:** Secondary outcome measures

Secondary outcomes	Measure description	Time point	
		Baseline	MU study
Child Outcomes			
Academic achievement			
Academic Ability	Wide Range Achievement Test (WRAT 4) – a psychometrically sound direct measure of reading and mathematical computation [63].	_	C
Academic Competence	Academic Competence (Social Skills Improvement System (SSIS)) - 7-item scale assessing the overall academic performance, motivation, reading and mathematical ability of the student in comparison other students in the classroom [64].	_	T
Behaviour			
ADHD Symptoms	ADHD Rating Scale IV - 18-item validated scale measuring the core symptoms of ADHD [58].	P, T	P, T
Problem Behaviours	Strengths and Difficulties Questionnaire (SDQ) *–* 25-item validated measure of behaviouraland emotional problems for childrenaged 4 to 16 years. There are 5 subscales; conduct problems, hyperactivity/inattention, emotional problems, peer problems, and prosocial behavior); a total problems score is derived from the first 4 subscales [65].	P, T	P, C, T
Social and Emotional Functioning (SEF)			
SEF Problems	SDQ Subscales *–* 5-item emotional and peer problems subscales [65].	P, T	P, C, T
Depression	Short Version Moods and Feelings Questionnaire (SMFQ) – 13-item subscale assessing depression symptoms in children and youth [66].	_	C
Bullying	Gatehouse Bullying Scale – 12-item scale measuring covert and overt victimisation [67].	_	C
Student Engagement			
Student Attitudes	Attitudes to school life – Motivation (5-items), Connectedness (5-items) and Commitment to school (5-items) scales, from the Victorian Attitudes to School Survey 2012, DEECD) [68].	-	C
School Attendance	School attendance – days absent over the preceding 3 months	_	P, C, T

C - Child P -Parent T-Teacher

### Risk and protective factors

We will measure a number of risk and protective factors that may impact on the transition outcomes of young people in the study. These risk and protective factors include child, family and school factors and are outlined in Table 2.

**Table 2:** Measures of risk and protective factors

Secondary outcomes	Measure description	Time point	
		Baseline	MU study
Child Risk Factors			
Quality of Life	Pediatric Quality of Life Inventory 4.0 - 23-item validated measure for children aged 2 to 18 years. Provides total, physical, and psychosocial health summary scores, with higher scores indicating better health-related quality of life [69].	P	_
Sleep problem severity	Primary caregiver report of child sleep problems (none, mild, moderate or severe) [70].	P	P
Difficulties withinitiating andmaintaining sleep	Sleep Disturbance Scale for Children (SDSC) –7-item subscale assessing disorders of initiating and maintaining sleep [71].	_	P
Sleep habits	Self-reported sleep habits – 2-items from the Longitudinal Study of Australian children about the amount and quality of sleep [72].	_	C
Comorbid Mental Health Problems	Anxiety Disorders Interview Schedule for DSM-IV - diagnostic interview assessing mental health disorders according to DSM-IV criteria [73].	P	_
Other Comorbidities	Learning difficulties or Autism Spectrum Disorder – parent-report of whether these conditions have been diagnosed by health professional*.*	P	P
Cognitive Functioning	Wechsler Abbreviated Scale of Intelligence™ (WASI™) – Provides an estimated general intellectual ability, based on two subsets, Vocabulary and Matrix Reasoning [74].	_	C
Working Memory	Wechsler Intelligence Scale for Children – Fourth Edition (WISC-IV) - Digit Span Forwards and Backwards subscale assessing short-term auditory memory [75].	_	C
Affective Reactivity Index (ARI)	Affective Reactivity Index (ARI) – 7-item measure of chronic irritability [76].	_	P,C
Substance Use	Substance Use – 6-items assessing alcohol, smoking and cannabis use ever and use in last 12 months. Questions previously used in the Victorian Adolescent Health and Wellbeing Survey [77].	_	C
Puberty	Puberty Scale – Self-rating scale of pubertal development from pre-pubertal through to post-pubertal (5 mins) [78].	_	C
Parent, Family and School Risk Factors			
Mental Health	Depression Anxiety Stress Scale - 21-item measure of adult mental health with clinical cut points for each of the three subscales of depression, anxiety and stress [79].	P	P
Family Functioning	Family Environment Scale – 9-items scale measuring family function/dysfunction [80].	P	_
School Environment	My School Variables – sector, type, year range, location and index of socio-educational advantage (SEA), [62].	_	D

*C* Child, *P* Parent, *T* Teacher, *D* Data Linkage

Socio-demographic variables are obtained via parent report at baseline and follow up. Important factors to be taken into account include: child age, gender, ADHD medication use, parent income, parent education and family status (partner living at home). The family socio-economic level will use the census-based Socio-Economic Indexes for Areas Disadvantage Index (SEIFA) [61] for the family postcode of residence.

We will link to school demographic data (e.g. school sector; government, non-government, type and location; metro, provincial, remote, very remote), available from the *My Schools* website [62] and ask teachers and parents about service usage (e.g. education support services use and education funding) for their child’s learning.

### Data analyses

Initially we will check for nonresponse bias, by comparing responders and non-responders on background characteristics obtained at baseline (outlined above).

Student and parent characteristics will be described using means and standard deviations for normally distributed continuous data and additionally medians and interquartile ranges for skewed continuous data; and percentages for categorical data.

To compare the performance of the cohort across the four outcome domains (academic achievement, social emotional, behaviour and school engagement) to the average performance of children within the state, data will be analysed using one-sample t-tests and 95 % confidence intervals. For example, we will compare academic achievement for the Moving Up children, defined as the mean NAPLAN standard score on each learning domain (measured from 0 to 1000), to the average achievement of children in the same school year in the state of Victoria, defined as the mean NAPLAN standard score for the state.

A bivariate analysis will be undertaken to determine potential covariates for the regression models from the risk and predictive factors shown in Table 2. Factors will be selected on the basis that they are significant at the level p < 0.1 in the bivariate analyses. A hierarchical multiple regression model will be used to estimate the adjusted effects of multiple factors on the children’s outcomes, examining predictors in groups i.e. child predictors then child and family/parent predictors, and lastly child plus family/parent plus school predictors.

### Sample size and power

We aim to have 150 families participate in the Moving Up study. Assuming NAPLAN results are available for 75 % of the cohort, power calculations show that the study is sufficiently powered to show meaningful differences in outcomes considering a p value of less than 0.05 as statistically significant. In comparison of NAPLAN test scores (primary outcome) - available for 115 students - to normative values, the study will provide 90 % power to detect a minimum difference of 0.3 standard deviations in either of the numeracy and reading outcomes, and 76 % power to detect a minimum difference of 0.25 standard deviations. For the multiple variable linear regression analysis, interview data for the 135 students participating will provide at least 80 % power to examine up to 5 independent predictor variables with a combined multiple correlation coefficient of R = 0.3.

## Discussion

A key milestone in a young person’s life is the transition from primary to secondary school. An ability to make a smooth and successful transition to secondary school is important for laying the foundations necessary to complete secondary school. The transition period can be stressful and poses challenges for most students, as they move from their familiar, often intimate primary school environment to an unfamiliar secondary environment. Making a successful transition to secondary school may protect young people from school disengagement and help frame life-long positive attitudes to learning. School dropout is linked with increased delinquent behaviour, crime, substance use and risk taking behaviour.

For students with ADHD who often struggle at school, this crucial transition period poses additional risks and challenges. Deficits associated with ADHD may make young people with ADHD particularly vulnerable during this period. A poor transition to high school may facilitate early disengagement with school and negatively influence attitudes to school and learning.

Few studies to date have focused on the high school transition period for students with ADHD, and most studies investigating educational outcomes for adolescents tend to focus on single domains of functioning (i.e. academic *or* social outcomes). A major strength of the ***Moving Up*** study is the focus on a number of transition outcomes across multiple and equally important domains of school functioning and to investigate what factors are associated with a poor versus good outcome. Little is known about how children with ADHD adjust to secondary school or what factors (e.g., symptom severity and comorbidity) are associated with better or worse high school transition outcomes. We are particularly concerned with identifying variables that can be modified to help promote positive school transition.

These findings will inform clinical practice, educators, parents and adolescents by providing a better understanding of the modifiable risk and protective factors associated with differing secondary school transition and early high school outcomes for young people with ADHD. Greater understanding of the challenges posed during this period will enable more targeted early interventions, services and resources to be developed to support these vulnerable and high-risk children, their families and schools.

### Ethics and dissemination

The study is approved by The Royal Children’s Hospital Melbourne Human Research Ethics Committee (33206). Approval to conduct research in Victorian schools has been granted by the Victorian Department of Education and Early Childhood Development (002202) and the Catholic Education Office (0009). Outcomes will be widely disseminated through conferences, seminars and peer-reviewed journals. This research is also being undertaken as part of NZ’s PhD.

## Abbreviations

ACARA: Australian Curriculum, Assessment and Reporting Authority
ADHD: Attention-Deficit/Hyperactivity Disorder
ATS: Attention to Sleep Study
DSM-IV: Diagnostic and Statistical Manual of Mental Disorders, Fourth Edition
DSM-5: Diagnostic and Statistical Manual of Mental Disorders, Fifth Edition
LSAC: Longitudinal Study of Australian Children
NAPLAN: The National Assessment Program - Literacy And Numeracy
NMS: National Minimum Standard
SDQ: Strengths and Difficulties Questionnaire
SS RCT: Sleeping Sound with ADHD Randomised Control Trial
VCAA: Victorian Curriculum and Assessment Authority
SES: Socio-Economic Status
